# Application of contrast-enhanced CT radiomics in prediction of early recurrence of locally advanced oesophageal squamous cell carcinoma after trimodal therapy

**DOI:** 10.1186/s40644-021-00407-5

**Published:** 2021-05-26

**Authors:** Sun Tang, Jing Ou, Jun Liu, Yu-ping Wu, Chang-qiang Wu, Tian-wu Chen, Xiao-ming Zhang, Rui Li, Meng-jie Tang, Li-qin Yang, Bang-guo Tan, Fu-lin Lu, Jiani Hu

**Affiliations:** 1 Sichuan Key Laboratory of Medical Imaging, Department of Radiology, Affiliated Hospital of North Sichuan Medical College, 63# Wenhua Road, 637000 Nanchong, Sichuan China; 2 Sichuan Key Laboratory of Medical Imaging, North Sichuan Medical College, Nanchong, Sichuan China; 3 Department of Radiology, Wayne State University, Detroit, Michigan USA

**Keywords:** Esophageal neoplasms, Carcinoma Squamous Cell, Tomography X-ray computed, Recurrence, Therapeutics

## Abstract

**Background:**

Early recurrence of oesophageal squamous cell carcinoma (SCC) is defined as recurrence after surgery within 1 year, and appears as local recurrence, distant recurrence, and lymph node positive and disseminated recurrence. Contrast-enhanced computed tomography (CECT) is recommended for diagnosis of primary tumor and initial staging of oesophageal SCC, but it cannot be used to predict early recurrence. It is reported that radiomics can help predict preoperative stages of oesophageal SCC, lymph node metastasis before operation, and 3-year overall survival of oesophageal SCC patients following chemoradiotherapy by extracting high-throughput quantitative features from CT images. This study aimed to develop models based on CT radiomics and clinical features of oesophageal SCC to predict early recurrence of locally advanced cancer.

**Methods:**

We collected electronic medical records and image data of 197 patients with confirmed locally advanced oesophageal SCC. These patients were randomly allocated to 137 patients in the training cohort and 60 in the test cohort. 352 radiomics features were extracted by delineating region-of-interest (ROI) around the lesion on CECT images and clinical signature was generated by medical records. The radiomics model, clinical model, the combined model of radiomics and clinical features were developed by radiomics features and/or clinical characteristics. Predicting performance of the three models was assessed with area under receiver operating characteristic curve (AUC), accuracy and F-1 score.

**Results:**

Eleven radiomics features and/or six clinical signatures were selected to build prediction models related to recurrence of locally advanced oesophageal SCC after trimodal therapy. The AUC of integration of radiomics and clinical models was better than that of radiomics or clinical model for the training cohort (0.821 versus 0.754 or 0.679, respectively) and for the validation cohort (0.809 versus 0.646 or 0.658, respectively). Integrated model of radiomics and clinical features showed good performance in predicting early recurrence of locally advanced oesophageal SCC for both the training and validation cohorts (accuracy = 0.730 and 0.733, and F-1score = 0.730 and 0.778, respectively).

**Conclusions:**

The integrated model of CECT radiomics and clinical features may be a potential imaging biomarker to predict early recurrence of locally advanced oesophageal SCC after trimodal therapy.

## Background

Oesophageal cancer is the eighth most common malignancies in the world [1], and squamous cell carcinoma (SCC) is the predominant histological type [2]. Locally advanced oesophageal SCC is defined as ≥ T2 or ≥ N1 carcinoma [3]. The survival rate of patients with locally advanced oesophageal SCC is extremely lower than that of patients with early oesophageal cancer, with the 5-year survival rate of only about 50 % [4–6]. Previous studies indicate that the 1-year recurrence rate of oesophageal SCC after appropriate treatment may range 40-70 % [7, 8]. As for early recurrence, it is defined as recurrence after surgery within 1 year, and appears as local recurrence, distant recurrence, and lymph node positive and disseminated recurrence [7, 9]. In order to improve the local survival, trimodal therapy including neoadjuvant chemotherapy, radiotherapy and surgery is often required in patients with locally advanced oesophageal cancer [10–12].

Currently, contrast-enhanced computed tomography (CECT) is commonly used for initial judging T and N staging of primary tumor [13], and ^18^ F-fluorodeoxyglucose-positron emission tomography CT (FDG-PET CT) is mainly used for judging M stage [14], but they cannot be used to predict early recurrence of locally advanced oesophageal SCC mainly based on the visually morphological and density features. Radiomics is a noninvasive tool to provide additional information by extracting high-throughput quantitative features from computed tomography (CT) images [15, 16]. It is reported that radiomics can be used to predict preoperative stages of oesophageal SCC [17], lymph node metastasis before operation [18], and 3-year overall survival of oesophageal SCC patients following chemoradiotherapy [19]. Although CT radiomics features have been applied and proved to be helpful for predicting early recurrence of hepatocellular carcinoma [20], there is no literature regarding the best way to use multiple imaging biomarkers as a predictive method to predict early recurrence of locally advanced oesophageal SCC. Thus, this study aimed to develop and validate novel models based on CT radiomics and clinical features of oesophageal SCC which can help predict early recurrence of locally advanced cancer after trimodal therapy so as to perform timely precise treatment to prevent the recurrence.

## Methods

### Patients

Ethical approval to perform this retrospective research was obtained from institution ethics committee of our hospital, and informed consent was waived. We retrospectively collected CT data and medical records of patients with locally advanced oesophageal SCC at our hospital from March 2016 to August 2018.

**Fig. 1:**
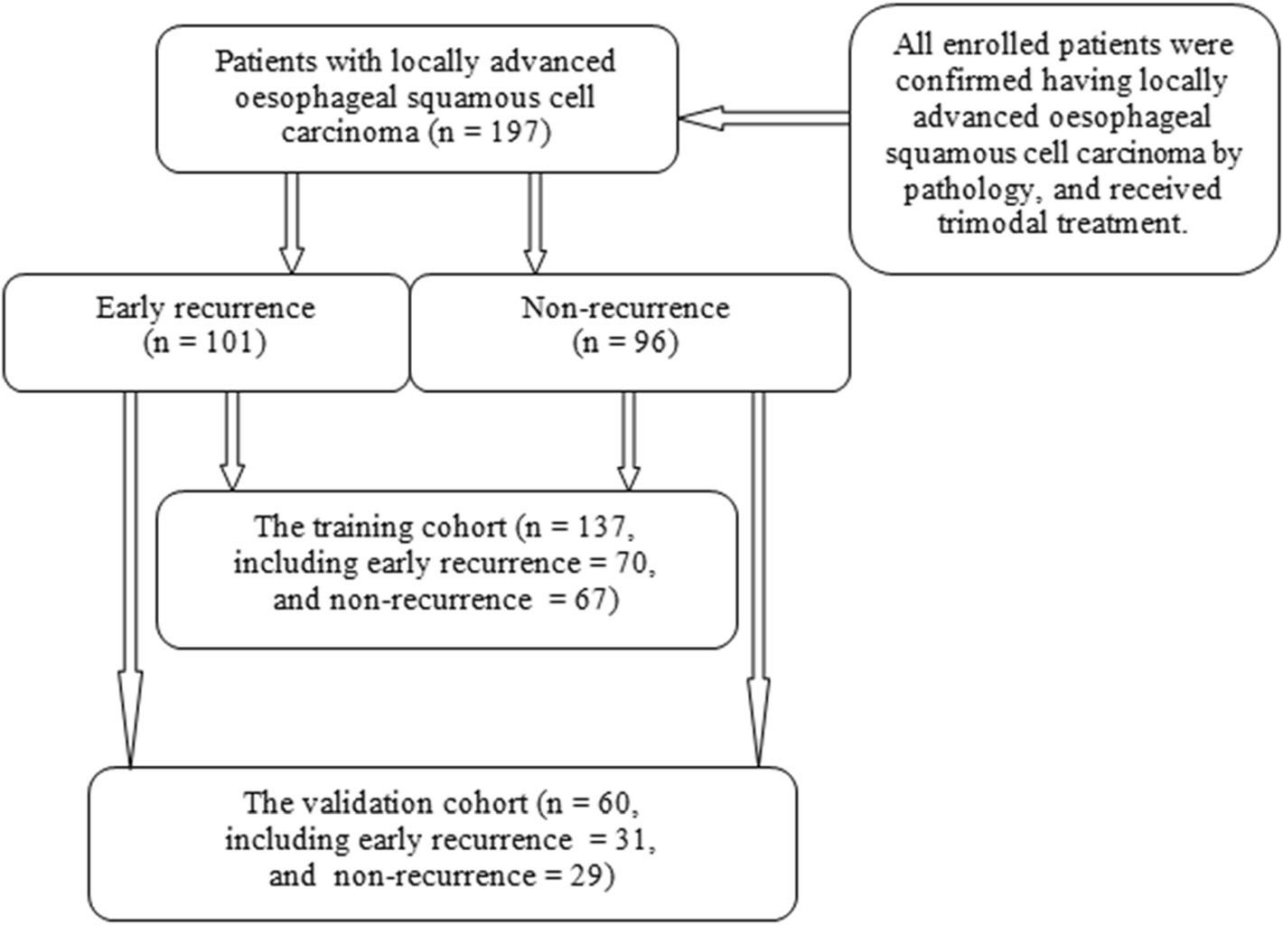
The flow chart for collecting patients

The enrolled patients needed to meet the following inclusion criteria: (1) oesophageal SCC patients underwent CECT and images with good quality were obtained within 2 weeks before the surgery; (2) the patients did not receive any tumor-related treatments (e.g., chemotherapy or radiotherapy) before undergoing the CT scans; (3) patients were confirmed no distant metastasis (M1) before surgery based on CT and/or PET-CT scans; (4) patients received standardized trimodal therapy, and were confirmed locally advanced oesophageal SCC and no residual tumor (R0) by postoperative pathology; and (5) patients were followed up for at least 1 year after trimodal therapy according to the latest edition of National Comprehensive Cancer Network (NCCN) guidelines [21] In our study, 230 patients met the previous inclusion criteria. Of this cohort, 158 patients were confirmed no distant metastasis by CT before surgery, and the remained 72 patients in which the status of distant metastasis could not be well confirmed by CT were finally confirmed no distant metastasis by PET-CT scans. On CT, T staging was judged by the depth of tumor invasion, and N staging was mainly determined by the morphological and enhancement characteristics of lymph nodes [22, 23]. As for the surgical indications [21], the criteria for unresectable oesophageal cancer were as follows: (1) cT4b tumours with involvement of the heart, great vessels, trachea, or adjacent organs including liver, pancreas, lung and spleen were considered unresectable; (2) oesophageal SCC with multi-station bulky lymphadenopathy was considered unresectable; or (3) oesophageal SCC with distant metastases including nonregional lymph nodes (stage IV) was unresectable. If the oesophageal SCC was not considered unresectable according to the previous NCCN guidelines, this tumour could be regarded resectable, and the patients received surgical treatment. The patients were excluded from this study according to the following exclusion criteria: (1) lost to follow up (*n* = 26); or (2) with incomplete clinicopathological information (*n* = 7). Consequently, 197 patients were enrolled into this study in total. According to the published report by Chen et al. [24], all participants were randomly assigned to the training and validation cohorts at the 7:3 ratio. The patient flowchart is shown in Fig. 1.

Archived clinical data, such as gender, age, histological grade, pT staging, pN staging and comprehensive staging were extracted by reviewing the medical records. Tumor staging was judged by the American Joint Committee on Cancer TNM Staging System Manual, 8th Edition [25].

### Follow-up

According to the latest edition of the NCCN guidelines [21], asymptomatic oesophageal SCC patients were followed up at 3 to 6 month intervals during the first 2 year, 6–12 month intervals until the fifth year, and then annually. If recurrence was suspected, patients underwent further clinical examinations including CT, ultrasonography, endoscopy, and ^18^FDG-PET CT for the confirmation of recurrence [9]. In our study, all patients underwent more than one year’s follow-up, and those with suspected recurrent lesions received cervical and thoracoabdominal CT, cerebral CT or magnetic resonance imaging, endoscopic biopsy, and even FDG-PET CT to diagnose the recurrence according to the published report [7–9].

### Image acquisition and retrieval procedure

All patients we collected underwent CECT scans with 128 multi-detector scanners (LightSpeed VCT, Ge Medical systems, USA). Patients who were suspected of having oesophageal cancer were required to drink 100–200 ml water before CT, which was used as oesophageal negative contrast material. During the CT examination, the patients were scanned in the supine position and hold a breath for 10–15 s to get satisfactory images. Following routine non-enhanced CT, CECT was performed a 25–30 s delay after intravenous injection of 1.5 mL/kg contrast material (Omnipaque, Iohexol, GE Healthcare, USA) at the rate of 3.0 mL/s with a pump injector (Vistron CT Injection System, Medrad, USA). The parameters for the CT scans were given as follows: 120 kV, 200 mAs, 0.5 s rotation time, detector collimation of 64 × 0.6 mm, pitch of 0.9, slice thickness of 5.0 mm, and matrix of 512 × 512 mm. The coverage of CT examination was from the base of skull to the middle of the left kidney. All CECT image data that were used for extracting features were obtained by picture archiving and communication system.

### Image segmentation and radiomics feature extraction

**Fig. 2:**
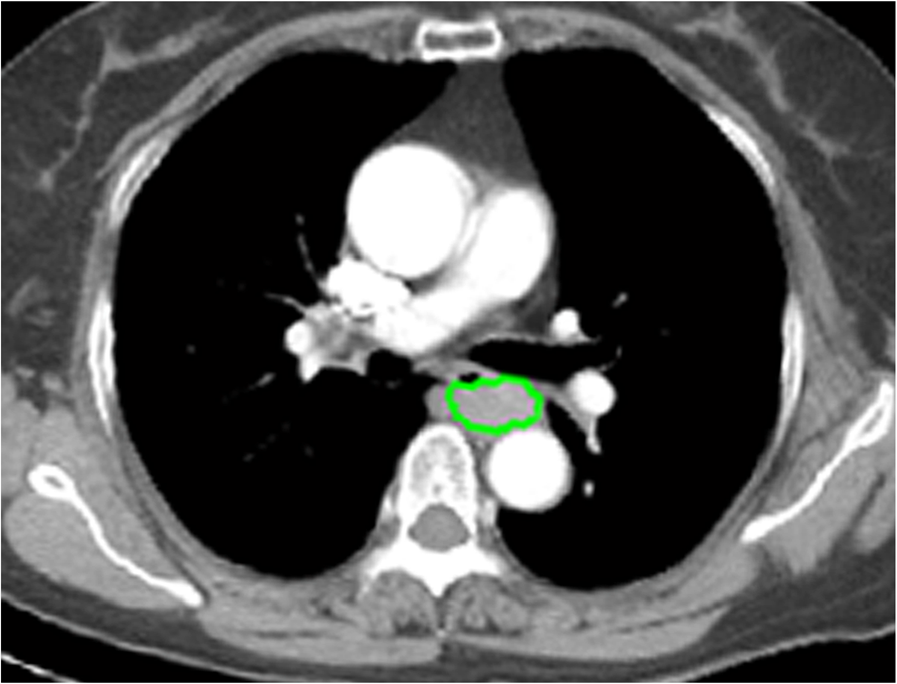
The outlines of oesophageal cancer are manually drawn slice by slice on contrast-enhanced CT data

The manual segmentation of tumor images (Fig. 2) was performed independently with IBEX (β1.0, http://bit.ly/IBEX_MDAnderson), an open source software, which ran on 64-bit MATLAB 2013Ra [26]. During delineating of the tumorous region-of-interest (ROI), we agreed that the thickness of oesophageal wall exceeding 5 mm was considered abnormal [27, 28]. When the tumor size exceeded 5 mm, we drew each ROI by taking both the tumour size and the abnormal contrast-enhanced area into consideration to determine the tumour area. Two radiologists (readers 1 (ST) and 2 (TWC), with 2 years and 22 years of medical imaging experience in the digestive system, respectively) were blinded to patients’ pathological results. When reader 1 and reader 2 disagreed with each other, they could reach consensus after discussion. For each ROI, the contour of locally advanced oesophageal SCC was delineated slice-by-slice around the lesion avoiding air, fat and bone. Features were extracted from IBEX, and included gray-level co-occurrence matrix (GLCM), gray-level run-length matrix (GLRLM), intensity histogram, and shape. There were 352 radiomics features in total for locally advanced oesophageal SCC to describe the tumor characteristics.

### Intra- and inter-observer agreements

In order to guarantee the reproducibility of results, we randomly selected consecutive 50 cases’ CECT data from all patients for intra- and inter-observer consistency testing. The intra-class correlation coefficient (ICC) was an evaluation index to assess intra- and inter-observer agreements. It meant good consistency when the ICC score was greater than 0.75. The ICC of intra-observer was measured by reader 1 drawing the outline of ROI twice within one week following the same steps according to the published report by Chen et al. [24]. In addition, reader 2 independently sketched the ROI of the lesion when reader 1 performed the first delineation, and the results of radiomics features extracted from these two delineations were compared to assess the inter-observer agreement based on the ICC value. Because of the nature of the variability resulting from the voxel-size and gray-level dependency [29], it was impossible that all radiomics signatures met the criteria for satisfactory agreement.

### Dimensionality reduction and radiomics feature selection

In order to avoid the curse of dimensionality and reduce the bias from radiomics features during modeling [24], we adopted the following methods to select the unique and optimal features in the training cohort.

First, all the radiomics features extracted from imaging data were processed to z-score normalization (mean is zero and variance is one) [30]: x _norm_ = $\frac{\text{x}-{\upmu }}{{\upsigma }}$, where x is the original feature value, µ is the mean value of this feature, and σ is the standard deviation.

Second, the independent samples *t* test or the Mann-Whitney U test was further used to select potential important features of training cohort. The radiomics features without statistical significance (*P*-value > 0.05) were excluded.

Finally, the least absolute shrinkage and selection operator method (LASSO) [29], capable for performing regression analysis on high-dimensional data, was used to select the core radiomics features for predicting early recurrence of oesophageal SCC. The 1-standard error of the minimum criteria (the 1-SE criteria, a simpler model) was used to adjust the regularization parameter (λ) for select feature using 10-fold cross-validation.

### Construction of the radiomics model

The optimal selected radiomics features and clinical characteristics were used to construct three predictive models through logistic regression, which was a classical machine learning method. The three predictive models were a radiomics model, a clinical model, and an integration of the radiomics and clinical features. The adjustment of the optimal predictive model followed the same process as described above. The confusion matrix calculated the area under receiver operating characteristic curve (AUC), accuracy, F-1score as well as sensitivity, specificity, positive predictive value (PPV), and negative predictive value (NPV) to evaluate training results and test results of the three predictive models.

### Statistical analysis

All data of radiomics characteristics were conducted through R (Version 3.4.4, https://www.r-project.org/). The “glmnet” package was used to perform LASSO regression, and “pROC” package was used to draw receiver operating characteristic (ROC) curves. Statistical analysis methods varied with the type of clinical characteristics. The normality of distribution was evaluated by Shapiro-Wilk test and the homogeneity of variance was tested by Bartlett test. Continuous variables, expressed as the mean or median, were compared by the independent *t*-test. The categorical variables were described in percentiles and compared using the Chi-square test or Fisher’s exact test. *P* < 0.05 meant statistical difference.

## Results

### Clinical characteristics

In our cohort of 197 patients with locally advanced oesophageal SCC, 101 patients recurred whereas the remaining 96 did not. In the 101 patients with recurred oesophageal SCC, the recurrence time was more than 3 months and less than or equal to 6 months after the treatments in 27 patients, more than 6 months and less than or equal to 9 months after the treatments in 39 patients, and more than 9 months and less than or equal to 12 months after the treatments in 35 patients. The clinical characteristics of locally advanced oesophageal SCC in the early recurrence and non–early recurrence dataset are recorded in Table 1. Among the six clinical features listed in Table 1, four clinical features including pT stage, pN stage, differentiation degree and pTNM stage indicated statistical differences between the early recurrence and non–early recurrence of oesophageal SCC (all *P*-values < 0.05) whereas age and gender were not statistically significant (all *P*-values > 0.05). All clinical features were used to establish a clinical model.

**Table 1:** Clinical features of early recurrence and non-early recurrence cohorts

	Early recurrence (*n* = 101)	Non-early recurrence (*n* = 96)	*P*-value
Median age (years )	62.5 (40 to 80)	63.0 (47 to 78)	0.665
Gender (%)			0.799
Male	72 (71.3)	70 (72.9)	
Female	29 (28.7)	26 (27.1)	
pT stage (%)			0.044^*^
T1	1 (1.0)	5 (5.2)	
T2	30 (29.7)	36 (37.5)	
T3	59 (58.4)	52 (54.2)	
T4a	11 (10.9)	3 (3.1)	
pN stage (%)			< 0.001^*^
N0	51 (50.5)	74 (77.1)	
N1	32 (31.7)	17 (17.7)	
N2	18 (17.8)	5 (5.2)	
Differentiation degree (%)			0.017^*^
Low	2 (1.9)	7 (7.3)	
Middle	55 (54.5)	35 (36.5)	
High	44 (43.6)	54 (56.2)	
pTNM stage (%)			< 0.001^*^
IB	7 (6.9)	20 (20.8)	
IIA	25 (24.8)	33 (34.4)	
IIB	15 (14.9)	21 (21.9)	
IIIA	8 (7.9)	4 (4.1)	
IIIB	46 (45.5)	18 (18.8)	

### Intra- and inter-observer agreements

**Fig. 3:**
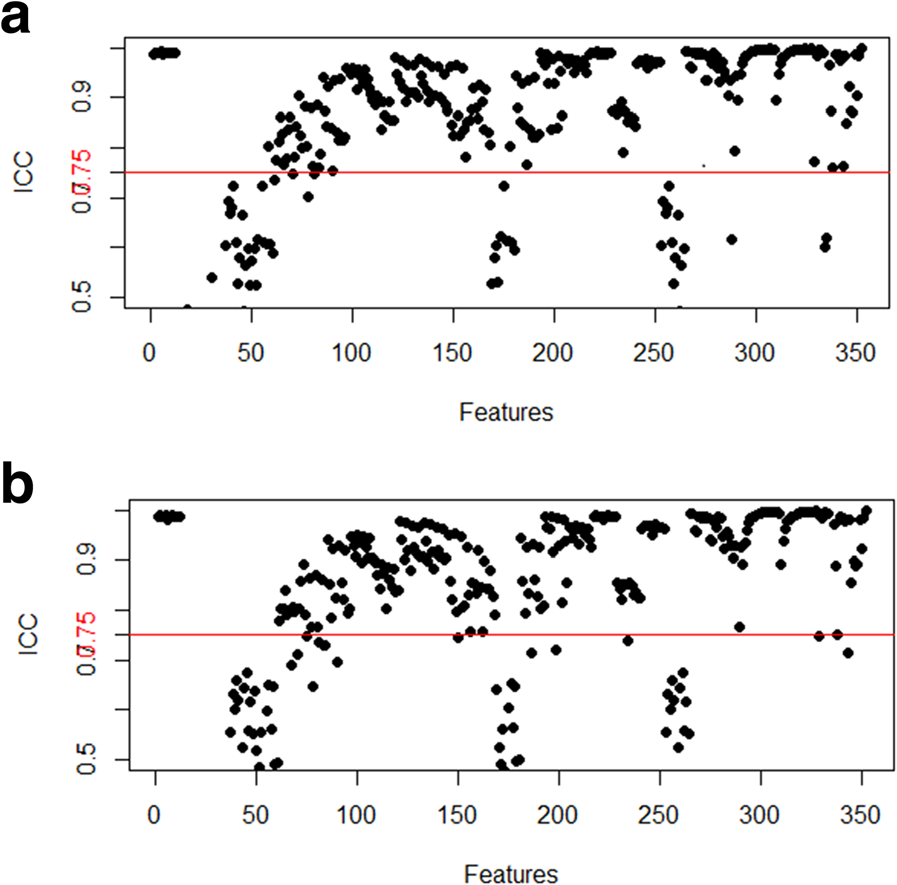
Characteristic stability evaluation with intra-observer (**a**) and inter-observer (**b**) inter-class correlation coefficient (ICC) for the extraction of CT radiomics features

For intra-observer agreement of CT radiomics feature extraction, there were 275 extracted features with ICC greater than 0.75 whereas ICC was not greater than 0.75 for 77 features (Fig. 3a). In terms of inter-observer agreement, there were 263 features with ICC greater than 0.75 while ICC was not greater than 0.75 for 89 features including the previous features with intra-observer ICC of not greater than 0.75 (Fig. 3b). After this assessment, the 89 features were excluded, and the remaining 263 features with intra- and inter-observer ICC values of greater than 0.75 were selected from the 352 extracted features for further analysis, and all results were derived from the feature extraction by reader 1.

### Dimensionality reduction and radiomics feature selection

**Fig. 4:**
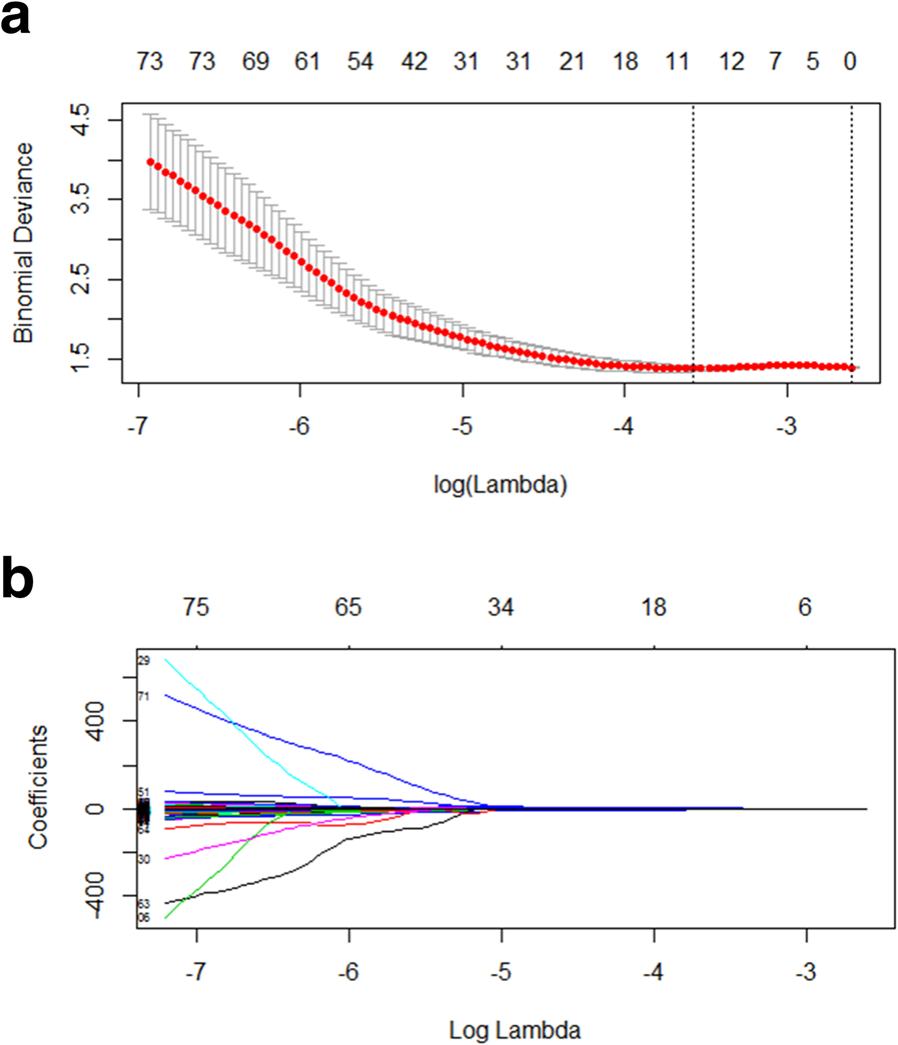
Feature selection using the least absolute shrinkage and selection operator (LASSO) regression. **a** Turning optimal parameter lambda (λ) using 10-fold cross-validation and minimum criterion in Lasso model. The left and right dashed lines represent the minimum criterion and the 1-standard error (1-SE) criterion, respectively. The 1-SE criterion has been applied. **b** Lasso coefficient profiles of the 263 radiomics features. The picture shows the optimal λ value of 0.025. 11 features with non-zero coefficients have been selected

The independent sample *t* test or Mann-Whitney U test showed that out of 263 features, 249 features were significantly different (all *P*-values < 0.05). Therefore, 249 features in total were used for LASSO regression, and 11 features were selected by LASSO (22.6:1 ratio) (Fig. 4a and b). These features included six texture features, four shape features, and one intensity histogram feature (Table 2).

**Table 2:** Selected features with descriptions

Feature category		Features of early recurrence vs. non-early recurrence
Texture features	GLCM	X45.7DifferenceEntropy
		X135.7Dissimilarity
		X0.1InformationMeasureCorr1
		X90.7InformationMeasureCorr2
		X45.7InverseVariance
	GLRCM	X90HighGrayLevelRunEmpha
Shape features		Compactness1
		Mass
		Orientation
		Roundness
Intensity histogram features	Intensity Histogram	Kurtosis
**Notes**: GLCM, Gray-level co-occurrence matrix; and GLRLM, Gray-level run-length matrix. GLCM features have been constructed by four directions (θ = 0°, 45°, 90°, and 135°) and three offsets (*d* = 1, 4, 7); and GLRLM features have been constructed by two directions (θ = 0°, 90°) and one offset (*d* = 1).		

### Construction of the radiomics model

**Fig. 5:**
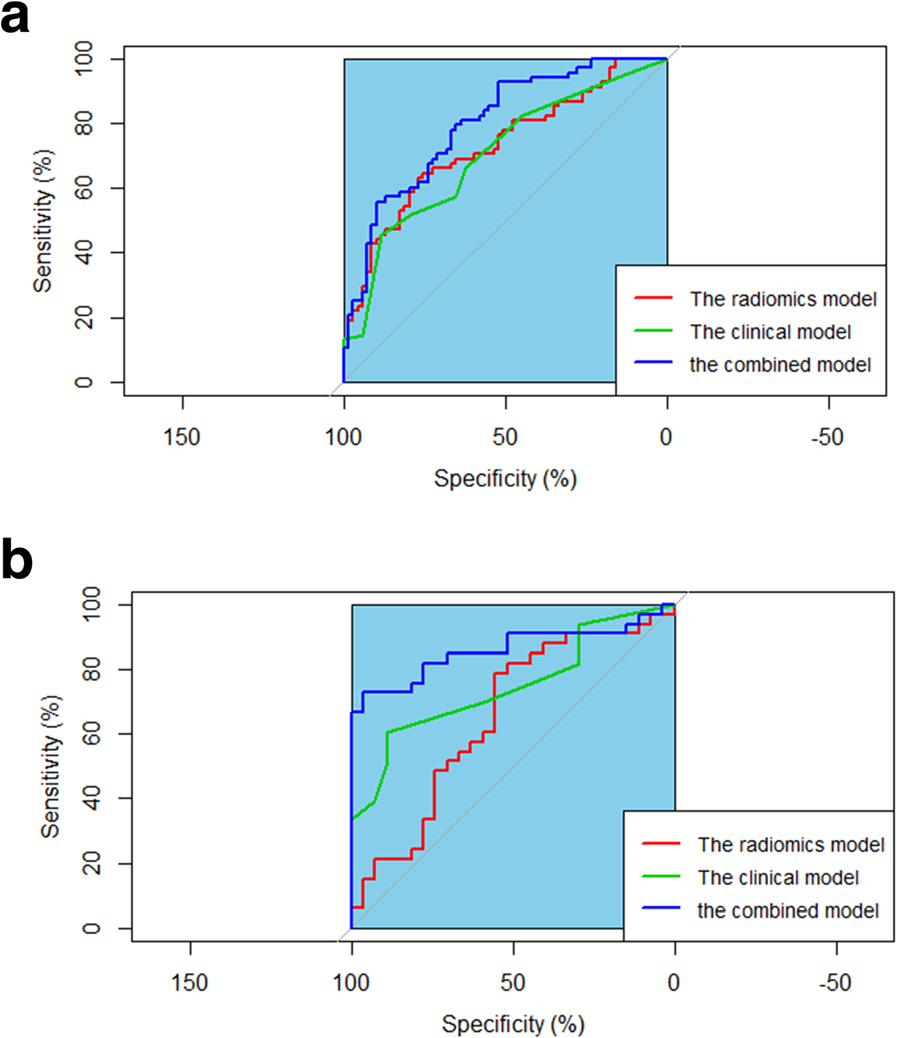
The receiver operating characteristic (ROC) curves show the performance of the radiomics model, the clinical model, and the combined model of radiomics and clinical features to predict early recurrence of locally advanced oesophageal squamous cell carcinoma after trimodal therapy in the training (**a**) and validation (**b**) cohorts

Through logistic regression, the 11 selected optimal radiomics features and six clinical features were applied to establish three prediction models in predicting early recurrence of locally advanced oesophageal SCC including a radiomics model, a clinical model, and a model that integrated radiomics and clinical characteristics. The most suitable model was selected by AUC, accuracy, and F-1 score together with sensitivity, specificity, PPV, NPV as shown in Table 3. Similarly in the pattern of ROC curves in the published literature [18], the ROC curves (Fig. 5a and b) visually indicated the integration model of radiomics and clinical features was superior to radiomics or clinical model in predicting early recurrence of locally advanced oesophageal SCC in the training cohort (AUC: 0.821 versus 0.754 or 0.679; and F1-score: 0.730 versus 0.690 or 0.671) and in the validation cohort (AUC: 0.809 versus 0.646 or 0.658; and F1-score: 0.778 versus 0.631 or 0.567, respectively). The DeLong test showed that the combined model of radiomics and clinical features was statistically better than the radiomics model or the clinical model in predicting early recurrence of locally advanced oesophageal SCC (all *P-*values < 0.05) whereas there were no statistical difference between the radiomics model and the clinical model (*P-*value > 0.05).

**Table 3:** The performance of the three constructed models to predict early recurrence of locally advanced oesophageal squamous cell carcinoma

Cohort	Prediction model	AUC	ACC	F1-score	Sen	Spe	PPV	NPV
The training cohort	Integrated model of radiomics and clinical features	0.821	0.730	0.730	0.735	0.725	0.725	0.735
	The radiomics model	0.754	0.682	0.690	0.694	0.662	0.694	0.662
	The clinical model	0.679	0.671	0.671	0.592	0.606	0.652	0.544
The validation cohort	Integrated model of radiomics and clinical features	0.809	0.733	0.778	0.761	0.720	0.718	0.762
	The radiomics model	0.646	0.630	0.631	0.655	0.613	0.613	0.655
	The clinical model	0.658	0.667	0.567	0.417	0.722	0.500	0.650
**Notes**: AUC, Area under the receiver operating characteristic curve; ACC, Accuracy; Sen, Sensitivity; Spe, Specificity; PPV, Positive predictive value; and NPV, Negative predictive value.								

## Discussion

Radiomics is a nascent image analysis method by extracting a large number of quantitative features to quantify tumor heterogeneity based on imaging data, which is of great significance for personalized oncology [31–33]. Radiomics has been attracting much attention because of its potential predictive power for treatment outcomes and cancer genetics in personalized medicine [34]. In this study, we have developed and validated a radiomics model based on the CECT images to predict the early recurrence of locally advanced oesophageal SCC after trimodal therapy.

Our study shows that pT stage, pN stage, pTNM stage and differentiation degree are independent clinical risk factors of early recurrence in locally advanced oesophageal SCC patients whereas age and gender are not among the six clinical characteristics which are used to establish the clinical model. As reported, the pT stage, pN stage and pTNM stage may be related to invasive depth of the tumor itself and the extent of lymphatic vessel invasion [7, 35]. As for differentiation degree, previous literature has shown that it is closely related to that tumor aggressiveness, but it is controversial whether the differentiation degree affects the prognosis of patients with oesophageal cancer [36, 37]. This study shows that when limited to locally advanced oesophageal SCC, the degree of differentiation has a certain relationship with early recurrence. However, the value of the clinical model in our study is limited in the prediction of early recurrence of locally advanced oesophageal SCC, with AUC values of 67.9 % in the training cohort and 65.8 % in the test cohort.

As shown in this paper, we selected 11 most unique features from all 352 features to build the CT radiomics model. The 11 features include texture features, intensity histogram features and shape features, which could be useful for assessing early recurrence of locally advanced oesophageal SCC. The six texture features and one intensity histogram feature include X45.7 Difference Entropy, X135.7 Dissimilarity, X0.1 Information Measure Corr1, X90.7 Information Measure Corr2, X45.7 Inverse Variance, X90 High Gray Level Run Empha, and Kurtosis. They can mainly represent the texture characteristics of tumors, which are highly associated with the heterogeneity and prognosis of the tumour [37, 38]. Shape features of Compactness1, Mass, Orientation, and Roundness provide the external features about the contours of the tumour. Our CT radiomics model shows a moderate performance in both the training cohort and the test cohort, with AUC values of 75.4 and 64.6 %, respectively, indicating that the clinical application of the radiomics model plays a certain role in capturing the intra-tumour heterogeneity information and alerting patients to potential recurrence [20].

The combined model obtains more satisfactory predictive results (with AUC values of 0.821 and 0.809 in training and test cohorts, respectively) compared with the other two models. It can be explained by the fact that the clinical and radiomics features complement each other to improve the predictive power of the combined model [16, 39, 40].

According to relevant literature [20, 41, 42], though radiomics has been applied to predict recurrence of many malignancies including liver cancer, rectal cancer and bladder cancer, methods for predicting the early recurrence of locally advanced oesophageal SCC after trimodal therapy based on CECT images are still lacking. We have built and verified the radiomics model to predict the early recurrence of locally advanced oesophageal SCC after trimodal therapy by taking the following measures to ensure the robustness. Firstly, as reported by Tan et al., it is reasonable to select oesophageal cancer lesions exceeding 5 mm for radiomics feature extraction [43]. We performed our radiomics study based on the referenced criteria that the thickness of oesophageal wall exceeding 5 mm was considered abnormal [27, 28]. As for radiomics analysis on the tumour exceeding 5 mm, we drew each ROI by taking both the tumour size and the abnormal contrast-enhanced area into consideration to determine the tumour area. Secondly, we used the reported CT scanning protocol to acquire the image data. According to the CT data acquisitions in the published radiomics research on oesophageal cancer [17], we deliberately selected early arterial phase images which were obtained 25–30 s after intravenous injection of the contrast material. Thirdly, to avoid overfitting the data, we set up test cohort to verify the ability of the radiomics model to predict early recurrence of locally advanced oesophageal SCC [20]. Last but not the least, univariate analysis, LASSO and Spearman correlation tests were used to guarantee the optimality and independence of each feature in the final model. Due to select optimal feature and modeling, we used 10-fold cross validation and stepwise regression to ensure the robustness of the model [29].

There are still some limitations in our retrospective study. First of all, this study is a relatively small sample and single-center study, and the applicability and universality of our findings needs to be further verified. Despite this limitation, our sample size was larger than that in the published reports focusing on radiomics study of oesophageal cancer [44, 45], suggesting that the relatively small sample in our study could still help obtain reliable results. Secondly, more studies are needed to demonstrate whether more clinical features can be considered in a comprehensive evaluation. Thirdly, previous studies have shown that thin-slice CT images can better display texture characteristics of tumours than thick-slice CT images [46]. It still remains to be verified whether thin slice CT images can increase the predictive power of the radiomics model.

## Conclusions

Our study explored the radiomics approach based on CECT images as a feasible method to predict early recurrence of locally advanced oesophageal SCC after trimodal therapy. The model by integrating CECT radiomics and clinical features can further improve the predictive performance of early recurrence of locally advanced oesophageal SCC when compared to the radiomics model or clinical model. We hope that our integrated model can be helpful for selecting the patients with high risk of early recurrence as early as possible to undergo effective personalized treatment and closer follow-up to prevent early recurrence of this cancer.

## Data Availability

Please contact the corresponding author for data requests.

## Abbreviations

SCC: Squamous cell carcinoma
CECT: Contrast-enhanced computed tomography
CT: Computed tomography
ROI: Region of interest
AUC: Area under the receiver operating characteristic curve
NCCN: National Comprehensive Cancer Network
GLCM: Gray-level co-occurrence matrix
GLRLM: Gray-level run-length matrix
ICC: Intra-class correlation coefficient
LASSO: Least absolute shrinkage and selection operator
ROC: Receiver operating characteristic
PPV: Positive predictive value
NPV: Negative predictive value
